# Insulin increases mRNA abundance of the amino acid transporter SLC7A5/LAT1 via an mTORC1‐dependent mechanism in skeletal muscle cells

**DOI:** 10.1002/phy2.238

**Published:** 2014-03-20

**Authors:** Dillon K. Walker, Micah J. Drummond, Jared M. Dickinson, Michael S. Borack, Kristofer Jennings, Elena Volpi, Blake B. Rasmussen

**Affiliations:** ^1^ Department of Nutrition and Metabolism University of Texas Medical Branch Galveston Texas; ^2^ Division of Rehabilitation Sciences University of Texas Medical Branch Galveston Texas; ^3^ Division of Epidemiology and Biostatistics Department of Preventive Medicine and Community Health University of Texas Medical Branch Galveston Texas; ^4^ Department of Internal Medicine University of Texas Medical Branch Galveston Texas; ^5^ Present address: Center for Translational Research in Aging and Longevity Texas A&M University College Station TX; ^6^ Present address: Department of Physical Therapy University of Utah Salt Lake City UT; ^7^ Present address: School of Nutrition and Health Promotion Arizona State University Phoenix AZ

**Keywords:** C2C12 myotubes, PAT1, rapamycin, SLC7A5, SNAT2

## Abstract

Amino acid transporters (AATs) provide a link between amino acid availability and mammalian/mechanistic target of rapamycin complex 1 (mTORC1) activation although the direct relationship remains unclear. Previous studies in various cell types have used high insulin concentrations to determine the role of insulin on mTORC1 signaling and AAT mRNA abundance. However, this approach may limit applicability to human physiology. Therefore, we sought to determine the effect of insulin on mTORC1 signaling and whether lower insulin concentrations stimulate AAT mRNA abundance in muscle cells. We hypothesized that lower insulin concentrations would increase mRNA abundance of select AAT via an mTORC1‐dependent mechanism in C2C12 myotubes. Insulin (0.5 nmol/L) significantly increased phosphorylation of the mTORC1 downstream effectors p70 ribosomal protein S6 kinase 1 (S6K1) and ribosomal protein S6 (S6). A low rapamycin dose (2.5 nmol/L) significantly reduced the insulin‐(0.5 nmol/L) stimulated S6K1 and S6 phosphorylation. A high rapamycin dose (50 nmol/L) further reduced the insulin‐(0.5 nmol/L) stimulated phosphorylation of S6K1 and S6. Insulin (0.5 nmol/L) increased mRNA abundance of SLC38A2/SNAT2 (*P* ≤ 0.043) and SLC7A5/LAT1 (*P* ≤ 0.021) at 240 min and SLC36A1/PAT1 (*P* = 0.039) at 30 min. High rapamycin prevented an increase in SLC38A2/SNAT2 (*P* = 0.075) and SLC36A1/PAT1 (*P* ≥ 0.06) mRNA abundance whereas both rapamycin doses prevented an increase in SLC7A5/LAT1 (*P* ≥ 0.902) mRNA abundance. We conclude that a low insulin concentration increases SLC7A5/LAT1 mRNA abundance in an mTORC1‐dependent manner in skeletal muscle cells.

## Introduction

Maintenance of muscle mass is crucial for functionality and quality of life in aging and disease. The balance of muscle mass is achieved by a larger net positive balance between muscle protein synthesis and breakdown. Muscle protein anabolism can be stimulated by amino acids (Drummond and Rasmussen 2008; Dickinson and Rasmussen 2011), insulin (Timmerman et al. 2010), and exercise (Fry et al. 2011; Walker et al. 2011). Insulin (in addition to amino acids and muscle contraction) is capable of increasing net muscle protein anabolism through mammalian/mechanistic target of rapamycin complex 1 (mTORC1) signaling in vivo (Timmerman et al. 2010) and in vitro (Proud 2002). Insulin‐induced activation of mTORC1 occurs via the phosphatidylinositol‐3‐kinase (PI3K)/Akt signaling pathway (Saltiel and Kahn 2001). Activation of mTORC1 results in phosphorylation of downstream effectors, p70 ribosomal protein S6 kinase 1 (S6K1) and eukaryotic initiation factor 4E‐binding protein 1 (4E‐BP1). S6K1 phosphorylates ribosomal protein S6 (rpS6) leading to increased translation of ribosomal and transcription factor mRNA resulting in protein translation initiation. Phosphorylation of 4E‐BP1 relieves the inhibitory action on eIF4E allowing the eIF4F translational initiation complex to form initiating protein translation.

Amino acid transporters (AATs) are ubiquitously expressed in many cell types and primarily function within the plasma membrane. Upon stimulation by insulin, activity, and recruitment of the AAT, sodium‐coupled neutral AAT 2 (SNAT2:SLC38A2) is enhanced (McDowell et al. 1998). SNAT2 mediates the Na^+^‐dependent transport of short‐chain amino acids and is recruited from an intracellular compartment to the plasma membrane in a PI3K‐dependent manner (Kashiwagi et al. 2009). Recent research suggests that specific AAT are capable of regulating mTORC1 signaling. For example, increasing intracellular glutamine concentrations via SNAT2 allows the antiport transporter, l‐type AAT (LAT1: which consists of a heterodimer of SLC7A5 and SLC3A2) to exchange leucine for glutamine thus activating mTORC1 (Baird et al. 2009). This coupling mechanism is critical for amino acid uptake and sensing upstream of mTORC1. Additionally, the AAT, proton‐assisted AAT (PAT1:SLC36A1) has been shown to be localized to the lysosomal membrane and facilitate mTORC1 activation (Ogmundsdottir et al. 2012). Given the potential regulatory role these AAT have on mTORC1 activation, understanding their regulation is necessary for maintaining muscle mass. Notably, mTORC1 has been shown to regulate LAT1 activity ultimately altering leucine uptake by the cell (Roos et al. 2007). Given the role insulin plays in mTOR activation, it is conceivable that insulin regulates select AATs. However, the effect of insulin on SLC38A2/SNAT2, SLC7A5/LAT1, SLC36A1/PAT1, and SLC7A1/CAT1 (cationic AAT1 – an AAT also linked to mTORC1 signaling; Huang et al. 2007), mRNA abundance and the involvement of mTORC1 signaling has not been determined in a muscle cell model using low insulin concentrations.

Clinical in vivo studies are a crucial starting point and provide valuable information; however, because the data are correlational, delineating precise mechanisms is limited. Murine C2C12 muscle myotubes are a widely used commercial cell line for studying nutrient regulation (Conejo and Lorenzo 2001; Shen et al. 2005; MacKenzie et al. 2009; Haegens et al. 2012). Using cell culture models provides an opportunity to conduct functional studies by altering specific media components to approximate human physiological levels. Therefore, the purpose of this study was to (1) compare different concentrations of insulin on mTORC1 signaling in C2C12 myotubes, and (2) determine the ability of low insulin concentrations to alter mRNA abundance of SLC38A2/SNAT2, SLC7A5/LAT1, SLC7A1/CAT1, and SLC36A1/PAT1 over a 4‐h period. We hypothesized that insulin (0.5 nmol/L) stimulates increases in AAT mRNA abundance and occurs via an mTORC1‐dependent mechanism.

## Experimental Procedures

### Cell culture

Murine C2C12 myoblasts were obtained from American Type Culture Collection and were cultured on 0.1% gelatin‐(Sigma‐Aldrich, St. Louis, MO) coated tissue cultureware in growth media (high‐glucose Dulbecco's modified Eagle medium supplemented with 10% fetal bovine serum, 50 U of penicillin/mL, 50 *μ*g of streptomycin/mL; Invitrogen, Carlsbad, CA) at 37°C in an atmosphere of 5% CO_2_/95% air. At ~90% confluency, differentiation medium (low‐glucose Dulbecco's modified Eagle medium supplemented with 2% horse serum, 50 U of penicillin/mL, 50 *μ*g of streptomycin/mL; Invitrogen, Carlsbad, CA) was added to cultures for 4–5 days to allow formation of multinucleated myotubes. Prior to experiments, myotubes were serum starved for 4 h followed by nutrient deprivation for 30 min in HEPES‐buffered saline (HBS, 20 mmol/L HEPES/Na, 140 mmol/L NaCl, 2.5 mmol/L MgSO_4_, 5 mmol/L KCl, and 1 mmol/L CaCl_2_; pH 7.4; Sigma‐Aldrich).

### Experimental design

**Figure 1:**
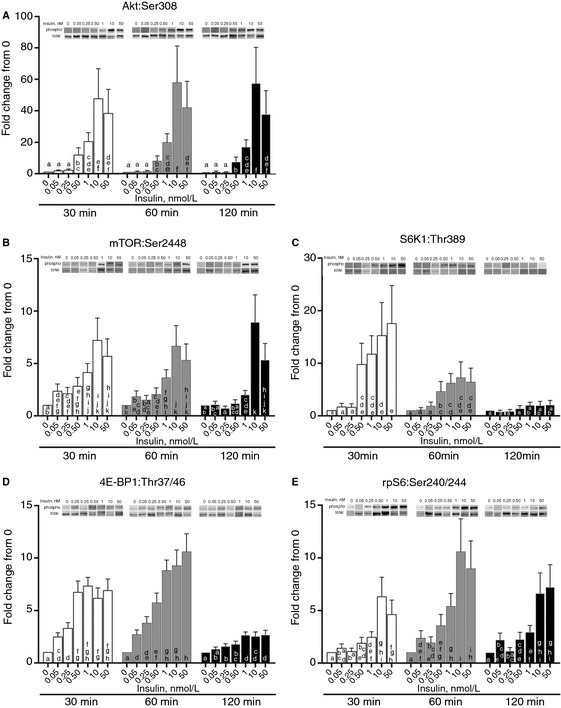
The effect of insulin concentrations on Akt and mTOR signaling. Different (0.05, 0.25, 0.5, 1, 10, and 50 nmol/L) concentrations of insulin (in HBS) were incubated with myotubes for 30, 60, and 120 min. Myotubes were lysed and protein extracts were analyzed using western blotting. (A) Phosphorylation status of Akt^Ser308^. ^abcdef^Columns with uncommon letters differ, *P* = 0.9921. (B) Phosphorylation status of mTOR^S^ ^er2448^. ^abcdefghijk^Columns with uncommon letters differ, *P* = 0.3123. (C) Phosphorylation status of S6K1^Thr389^. ^abcde^Columns with uncommon letters differ, *P* = 0.0376. (D) Phosphorylation status of 4E‐BP1^Thr37/46^. ^abcdefgh^Columns with uncommon letters differ, *P* < 0.0001. (E) Phosphorylation status of ribosomal protein S6^Ser240/244^. ^abcdefghi^Columns with uncommon letters differ, *P* = 0.775. Resulting images are displayed from a representative experiment above each graph. For arrangement of samples in gels for electrophoresis, all time points for two samples were run on a single gel. Thus, all samples were not run on a single gel/blot. Data are mean ± SEM and are presented as phospho/total made relative to baseline.

**Figure 2:**
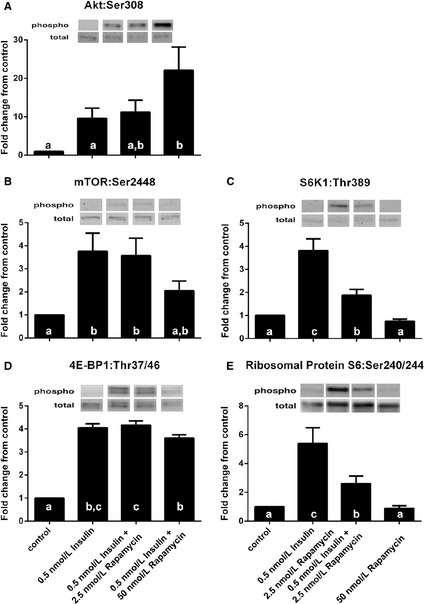
The effect of rapamycin on 0.5 nmol/L insulin‐stimulated increases in Akt and mTORC1 signaling. Cells receiving rapamycin were pretreated with either 2.5 or 50 nmol/L rapamycin (in HBS) for 30 min prior to receiving insulin. Then insulin (0.5 nmol/L) in HBS was incubated with cells with or without 2.5 or 50 nmol/L rapamycin for 30 min. Myotubes were lysed and protein extracts were analyzed using western blotting. ^abc^Columns with uncommon letters differ, main effect of treatment, (A) *P* = 0.0246 for Akt^Ser308^; (B) *P* = 0.0240 for mTOR^S^ ^er2448^; (C) *P* = 0.0004 for S6K1^Thr389^; (D) *P* < 0.0001 for 4E‐BP1^Thr37/46^; (E) *P* = 0.0019 for ribosomal protein S6^Ser240/244^. Resulting images are displayed from a representative experiment above each graph. For arrangement of samples in gels for electrophoresis, samples from two experiments were run on a single gel. Thus, all samples were not run on a single gel/blot. Data are mean ± SEM and are presented as phospho/total made relative to baseline.

**Figure 3:**
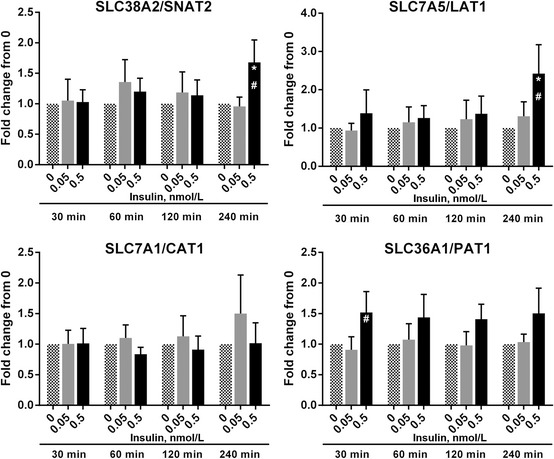
The effect of 0.5 nmol/L insulin on amino acid transporter mRNA abundance myotubes received 0, 0.05, or 0.5 nmol/L concentrations of insulin (in HBS) for 30, 60, 120, and 240 min. Myotubes were lysed, RNA isolated, cDNA synthesized, and analyzed using real‐time qPCR for mRNA abundance of (A) SLC38A2/SNAT2, (B) SLC7A5/LAT1, (C) SLC7A1/CAT1, (D) SLC36A1/PAT1. *Different from 0, *P* < 0.05. ^#^Different from 0.05, *P* < 0.05. Data are mean ± SEM and calculated using the ∆∆Ct method with GAPDH used as the reference gene.

**Figure 4:**
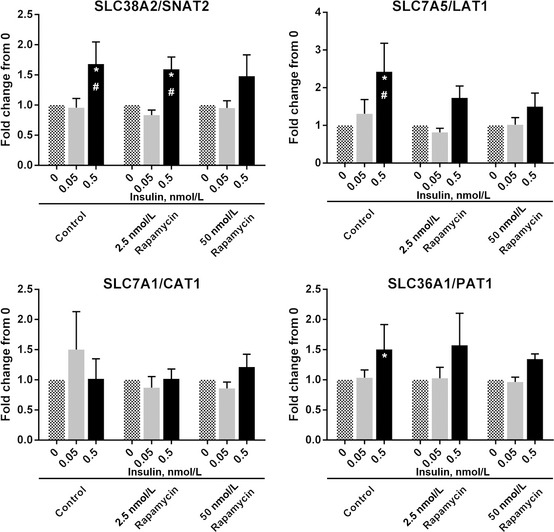
The effect of rapamycin on 0.5 nmol/L insulin‐stimulated increase in amino acid transporter mRNA abundance. Cells receiving rapamycin were pretreated with either 2.5 or 50 nmol/L rapamycin (in HBS) for 30 min prior to receiving insulin. Then 0, 0.05, or 0.5 nmol/L insulin in HBS was incubated with cells with or without 2.5 or 50 nmol/L rapamycin for 240 min. Myotubes were lysed, RNA isolated, cDNA synthesized, and analyzed using real time qPCR for mRNA abundance of (A) SLC38A2/SNAT2, (B) SLC7A5/LAT1, (C) SLC7A1/CAT1, (D) SLC36A1/PAT1. *Different from 0, *P* < 0.05. ^#^Different from 0.05, *P* < 0.05. Data are mean ± SEM and calculated using the ∆∆Ct method with GAPDH used as the reference gene.

In experiment 1 (Fig. 1), myotubes were incubated with 0.05, 0.25, 0.5, 1, 10, and 50 nmol/L insulin concentrations for 30, 60, and 120 min. For experiment 2 (Fig. 2), myotubes were incubated with 0.5 nmol/L insulin for 30 min with and without a low (2.5 nmol/L) and high (50 nmol/L) dose of rapamycin. Rapamycin was preincubated with myotubes 30 min prior to receiving insulin. The rapamycin concentration of 2.5 nmol/L (low) was used to represent the peak concentration recorded in plasma when 16 mg rapamycin was administered to subjects (Dickinson et al. 2011) and 50 nmol/L (high) was used to represent a higher dose used in cell culture experiments (Guertin et al. 2006; Wen et al. 2013). For experiment 3 (Fig. 3), 0.05 and 0.5 nmol/L insulin were incubated with myotubes for 30, 60, 120, and 240 min. For experiment 4 (Fig. 4), myotubes were incubated with or without 2.5 or 50 nmol/L rapamycin 30 min prior to incubation with 0.05 or 0.5 nmol/L insulin for 240 min. In all experiments, myotubes incubated with 0 insulin and/or 0 rapamycin were included and referred to as baseline.

### RNA isolation and real‐time qPCR

Following treatments, myotubes were rinsed with PBS three times and myotubes were scrapped in 1 mL TRI Reagent for RNA isolation. The RNA was separated into an aqueous phase using 0.2 mL chloroform and precipitated from the aqueous phase using 0.5 mL of isopropanol. The resultant RNA pellet was washed with 1 mL of 75% ethanol, air dried, and then suspended in a known amount of nuclease‐free water. RNA concentration was determined using the NanoDrop 2000 spectrophotometer (Thermo Fisher Scientific, Wilmington, DE). A total of 1 *μ*g of RNA was reverse transcribed into cDNA according to the manufacturer's protocol (iScript; BioRad, Hercules, CA). Real‐time qPCR was carried out with an iQ5 multicolor Real‐Time PCR cycler (BioRad) using SYBR green fluorescence (iQ SYBR green supermix; BioRad). Primer sequences are presented in Table 1. GAPDH was utilized as a housekeeping gene and relative fold changes were determined from the Ct values using the ∆∆Ct method.

**Table 1:** Mouse primer sequences used for real‐time qPCR.

Protein	Gene	Accession #		Primer sequence (5′ to 3′)
GAPDH	*GAPDH*	NM_008084	Forward	CCAGCAAGGACACTGAGCAAGA
			Reverse	TCCCTAGGCCCCTCCTGTTAT
SNAT2	*SLC38A2*	NM_175121	Forward	GGCATTCAATAGCACCGCAG
			Reverse	ACGGAACTCCGGATAGGGAA
LAT1	*SLC7A5*	NM_011404	Forward	CTTCGGCTCTGTCAATGGGT
			Reverse	TTCACCTTGATGGGACGCTC
CAT1	*SLC7A1*	NM_007513	Forward	GTCTATGTCCTAGCCGGTGC
			Reverse	GAGCCTAGGAGACTGGTGGA
PAT1	*SLC36A1*	NM_153139	Forward	CCGCTACCATGTCCACACAG
			Reverse	GGCCACGATACCAATCACCA

### Protein isolation and western blotting

Following treatments, myotubes were rinsed with PBS three times. Myotubes were scrapped in ice‐cold extraction buffer (50 mmol/L Tris‐HCl, 250 mmol/L mannitol, 50 mmol/L NaF, 5 mmol/L Na pyrophosphate, 1 mmol/L EDTA, 1 mmol/L EGTA, 1% Triton X‐100, 1 mmol/L DTT, 1 mmol/L benzamidine, 0.1 mmol/L PMSF, 5 *μ*g/mL soybean trypsin inhibitor, pH 7.4) then snap frozen in liquid nitrogen and thawed to facilitate cell lyses. Cell lysates were vortexed three times and sonicated for 15 sec. Protein concentrations were determined using a Bradford Protein Assay (Smartspec Plus, Bio‐Rad, Hercules, CA). Cell lysates were diluted (1:1) in a 2× sample buffer mixture (125 mmol/L Tris, pH 6.8, 25% glycerol, 2.5% SDS, 2.5% *β*‐mercaptoethanol, and 0.002% bromophenol blue) and then boiled for 3 min at 100°C. Equal amounts of total protein (7 *μ*g) were loaded into each lane and the samples were separated by electrophoresis at 150 V for 60 min on a 7.5% or 15% polyacrylamide gel (Criterion, Bio‐Rad). Each sample was loaded in duplicate and each gel contained an internal loading control and molecular weight ladder (Precision Plus, Bio‐Rad).

Following electrophoresis, protein was transferred to a polyvinylidene difluoride membrane (Bio‐rad) at 50 V for 60 min. Blots were blocked in 5% nonfat dry milk for 1 h and then incubated with primary antibody overnight at 4°C (see below). The following morning, blots were incubated in secondary antibody for 1 h at room temperature. Blots were then incubated in a chemiluminescent solution (ECL plus, Amersham BioSciences, Piscataway, NJ) for 5 min and optical density measurements were made using a digital imager (ChemiDoc, Bio‐Rad) and densitometric analysis was performed using Quantity One 4.5.2 software (Bio‐Rad). Membranes containing phospho‐detected proteins were stripped of primary and secondary antibodies using Restore Western Blot Stripping buffer (Pierce Biotechnology, Rockford, IL) and were reprobed for total protein with the specific antibody of interest. Phospho and total density values were normalized to the internal loading control and the phospho:total protein ratios were determined. Immunoblot data are expressed as phospho divided by total protein and adjusted to represent fold change from baseline (0 insulin and/or 0 rapamycin).

### Antibodies

The phospho and total antibodies used for immunoblotting were purchased from Cell Signaling (Beverly, CA): phospho‐Akt (Ser^308^; 1:1000), phospho‐mTOR (Ser^2448^; 1:500), phospho‐S6K1 (Thr^389^;1: 500), phospho‐4E‐BP1 (Thr^37/46^; 1:1000), and phospho‐rpS6 (Ser^240/244^; 1:250). Total protein was detected for Akt (1:1000), mTOR (1:500), S6K1 (1:500), 4E‐BP1 (1:1000), and rpS6 (1:250). Anti‐rabbit IgG HRP‐conjugated secondary antibody was purchased from Amersham Bioscience (1:2000).

### Statistical analysis

Data were analyzed using the MIXED procedure of SAS System for Windows Release 9.3 (SAS Institute Inc., Cary, NC). Data were analyzed as a completely randomized design. For insulin and time titration experiments for protein expression data, the model contained the effects of insulin and time, along with their interaction; to account for nonconstant variance in the data, all values were log transformed before being modeled. Each representative was modeled as a random blocking factor with a Kenwood‐Rodgers degrees of freedom adjustment. For insulin × rapamycin experiments for protein expression data and AAT mRNA abundance data, the model contained the effects of insulin and rapamycin, and along with an interaction term. Each representative was modeled as a random blocking factor. For insulin and time titration experiments for AAT mRNA abundance data, the model contained the effects of insulin and time, and along with an interaction term. Each representative was modeled as a random blocking factor with a Kenwood‐Rodgers degrees of freedom adjustment. Treatment means were computed using the LSMEANS option, and pairwise *t*‐tests were used to separate means and significance was determined at *P* < 0.05. All data are presented at the original scale with standard errors.

## Results

### Insulin increases phosphorylation of Akt and mTORC1 signaling

To investigate whether varying concentrations of insulin would stimulate Akt and mTORC1 signaling, increasing concentrations of insulin were incubated with C2C12 myotubes for 30, 60, and 120 min. Insulin concentrations of 0.05 nmol/L and 0.25 nmol/L did not alter phosphorylation of Akt relative to baseline across all time points (Fig. 1A; effect of insulin, *P* < 0.001). However, incubation of myotubes with 0.5 and 1 nmol/L insulin significantly increased Akt phosphorylation above baseline, 0.05, and 0.25 nmol/L insulin and incubation with 10 and 50 nmol/L insulin significantly increase Akt phosphorylation above baseline, 0.05, 0.25, 0.5, and 1 nmol/L insulin. No significant insulin × time interaction was detected (*P* = 0.99). Phosphorylation of mTOR was elevated at 30 and 60 min compared to 120 min (Fig. 1B; effect of time, *P* = 0.003). Incubation of myotubes with 0.05, 0.25, 0.5, and 1 nmol/L insulin increased mTOR phosphorylation above baseline whereas 10 and 50 nmol/L insulin increased mTOR phosphorylation above baseline, 0.05, 0.25, 0.5, and 1 nmol/L (effect of insulin, *P* < 0.001). No significant insulin × time interaction was detected (*P* = 0.312). Incubation of myotubes with 0.05 and 0.25 nmol/L insulin did not change S6K1 phosphorylation relative to baseline. However, 0.5, 1, 10, and 50 nmol/L insulin increased S6K1 phosphorylation above baseline, 0.05, and 0.25 nmol/L insulin at 30 min, to a lesser extent at 60 min, but not at 120 min (Fig. 1C; insulin × time interaction, *P* = 0.038). Phosphorylation of ribosomal protein S6 was greatest at 60 min compared to 30 and 120 min (Fig. 1E; effect of time, *P* = 0.0003). Incubation of myotubes with 0.5 and 1 nmol/L insulin increased ribosomal protein S6 phosphorylation above baseline, 0.05, and 0.25 whereas 10 and 50 nmol/L insulin increased ribosomal protein S6 phosphorylation above all other insulin concentrations (effect of insulin, *P* = 0.0003). At 30 and 60 min of incubation, 0.05 and 0.25 nmol/L insulin increased phosphorylation of 4E‐BP1 above baseline and 0.50, 1, 10, and 50 nmol/L insulin increased phosphorylation of 4E‐BP1 above baseline and 0.05 (and 0.25 nmol/L at 30 min) whereas only 1, 10, and 50 nmol/L insulin increased 4E‐BP1 phosphorylation above baseline at 120 min (Fig. 1D; insulin × time interaction, *P* < 0.001).

### Rapamycin inhibits insulin‐induced mTORC1 signaling

To determine whether rapamycin can reduce insulin (0.5 nmol/L; which approximates human postprandial insulin concentrations) induced upregulation of mTORC1 signaling, myotubes were incubated with a low (2.5 nmol/L) and high (50 nmol/L) concentration of rapamycin for 30 min prior to the addition of 0.5 nmol/L insulin to cultures. Incubation of myotubes with 0.5 nmol/L insulin and 0.5 nmol/L insulin plus low rapamycin did not change Akt phosphorylation above baseline (Fig. 2A; *P* ≥ 0.076) whereas the addition of high rapamycin with 0.5 nmol/L insulin increased Akt phosphorylation above baseline and insulin alone (*P* ≤ 0.036; effect of treatment, *P* = 0.025). mTOR phosphorylation was increased above baseline with 0.5 nmol/L insulin and 0.5 nmol/L insulin plus low rapamycin (*P* ≤ 0.013); however, incubation with 0.5 nmol/L insulin plus high rapamycin did not change phosphorylation of mTOR relative to baseline, 0.5 nmol/L insulin and 0.5 nmol/L insulin plus low rapamycin (Fig. 2B; *P* ≥ 0.054; effect of treatment, *P* = 0.024). Phosphorylation of S6K1 was increased above baseline by 0.5 nmol/L insulin (*P* = 0.0002), and by 0.5 nmol/L insulin plus low rapamycin (*P* = 0.0401) whereas S6K1 phosphorylation was not different than baseline when 0.5 nmol/L insulin plus high rapamycin were added (Fig. 2C; *P* = 0.544; effect of treatment, *P* = 0.0004). S6K1 phosphorylation was less with 0.5 nmol/L insulin plus low rapamycin compared to 0.5 nmol/L insulin (*P* = 0.001) and was less with 0.5 nmol/L insulin plus high rapamycin compared to 0.5 nmol/L insulin plus low rapamycin (*P* = 0.017). Phosphorylation of ribosomal protein S6 was increased above baseline by 0.5 nmol/L insulin (*P* = 0.0007) and by 0.5 nmol/L insulin plus low rapamycin (*P* = 0.042) whereas ribosomal protein S6 phosphorylation was not different than baseline when 0.5 nmol/L insulin plus high rapamycin were added (Fig. 2E; *P* = 0.936; effect of treatment, *P* = 0.002). Ribosomal protein S6 phosphorylation was less with 0.5 nmol/L insulin plus low rapamycin compared to 0.5 nmol/L insulin (*P* = 0.008) and was less with 0.5 nmol/L insulin plus high rapamycin compared to 0.5 nmol/L insulin plus low rapamycin (*P* = 0.038). Phosphorylation of 4E‐BP1 was increased by 0.5 nmol/L insulin (*P* < 0.001), 0.5 nmol/L insulin plus low rapamycin (*P* < 0.0001), and 0.5 nmol/L insulin plus high rapamycin (*P* < 0.001) whereas 4E‐BP1 phosphorylation by 0.5 nmol/L insulin plus low rapamycin was not different compared to 0.5 nmol/L insulin (*P* = 0.636) and was decreased by 0.5 nmol/L insulin plus high rapamycin compared to 0.5 nmol/L insulin plus low rapamycin (Fig. 2D; *P* = 0.032; effect of treatment, *P* < 0.001).

### Insulin increases mRNA abundance of SLC38A2/SNAT2, SLC7A5/LAT1, and SLC36A1/PAT1, but not SLC7A1/CAT1

Next, we wanted to determine if 0.5 nmol/L insulin would increase mRNA abundance of specific AAT in C2C12 myotubes. When myotubes were incubated with 0.05 and 0.5 nmol/L insulin, no changes in the mRNA abundance of SLC38A2/SNAT2 (Fig. 3A) and SLC7A5/LAT1 (Fig. 3B) were noted at 30, 60, and 120 min (insulin × time interaction, *P* = 0.529 for SLC38A2/SNAT2 and *P* = 0.549 for SLC7A5/LAT1); however, at 240 min SLC38A2/SNAT2 (*P* ≤ 0.043) and SLC7A5/LAT1 (*P* ≤ 0.021) were increased by 0.5 nmol/L insulin as compared to baseline and 0.05 nmol/L insulin. Across all times, 0.5 nmol/L insulin increased SLC7A5/LAT1 mRNA abundance relative to baseline and 0.05 nmol/L insulin (effect of insulin, *P* = 0.031). No differences were noted in SLC7A1/CAT1 mRNA abundance (Fig. 3C; insulin × time interaction, *P* = 0.897). Insulin (0.5 nmol/L) increased SLC36A1/PAT1 mRNA abundance at 30 min relative to 0.05 nmol/L insulin (*P* = 0.039), tended to increase SLC36A1/PAT1 at 30 min relative to baseline (*P* = 0.077), and tended to increase SLC36A1/PAT1 at 240 min relative to baseline (*P* = 0.086) and 0.05 nmol/L insulin (Fig. 3D; *P* = 0.109; insulin × time interaction, *P* = 0.998). Across all time points, 0.5 nmol/L insulin increased SLC36A1/PAT1 mRNA abundance relative to baseline and 0.05 nmol/L insulin (effect of insulin, *P* = 0.002).

### Rapamycin inhibits the insulin‐induced increase in SLC7A5/LAT1 mRNA abundance

We next wanted to determine if incubation of myotubes with a low and high concentration of rapamycin would reduce the increases in AAT mRNA abundance demonstrated by 0.5 nmol/L insulin in C2C12 myotubes at 4 h. Since the mRNA abundance of SLC38A2/SNAT2 and SLC7A5/LAT1 were increased and SLC36A1/PAT1 was numerically increased at 4 h, we considered this time point to be optimal for stimulation by insulin. Incubation of myotubes with 0.5 nmol/L insulin increased SLC7A5/LAT1 mRNA abundance relative to baseline (*P* = 0.001) and 0.05 nmol/L insulin (*P* = 0.014) whereas this increase was not seen when incubating myotubes with 0.5 nmol/L insulin plus low rapamycin (*P* = 0.09) and with high rapamycin (Fig. 4B; *P* ≥ 0.226; insulin × rapamycin interaction, *P* = 0.026). Similarly, SLC7A5/LAT1 mRNA abundance with 0.5 nmol/L insulin was not different than 0.5 nmol/L insulin plus low or high rapamycin (*P* ≥ 0.11). Incubation of myotubes with 0.5 nmol/L insulin increased SLC38A2/SNAT2 mRNA abundance relative to baseline (*P* = 0.031) and 0.05 nmol/L insulin (*P* = 0.027) with and without low rapamycin (Fig. 4A; insulin × rapamycin interaction, *P* = 0.015). However, SLC38A2/SNAT2 mRNA abundance was unchanged in myotubes when incubated with 0.5 nmol/L insulin plus high rapamycin relative to baseline (*P* = 0.075) and 0.5 nmol/L insulin (*P* = 0.493). SLC7A1/CAT1 mRNA abundance was not affected by the addition of 0.05 and 0.5 nmol/L (*P* ≥ 0.092) insulin with and without low and high rapamycin (*P* ≥ 0.182; Fig. 4C; insulin × rapamycin interaction, *P* = 0.589). SLC36A1/PAT1 mRNA abundance was increased by 0.5 nmol/L insulin relative to baseline (*P* = 0.042) and was not different with 0.5 nmol/L insulin relative to 0.05 nmol/L insulin (*P* = 0.105) whereas 0.5 nmol/L insulin incubated with low rapamycin tended to increase SLC36A1/PAT1 mRNA abundance relative to baseline (*P* = 0.063). No changes were noted when 0.5 and 0.05 nmol/L insulin were incubated with high rapamycin relative to baseline (*P* ≥ 0.3) and 0.5 nmol/L insulin alone (*P* ≥ 0.587; Fig. 4D; insulin × rapamycin interaction, *P* = 0.221).

## Discussion

The coupling of insulin signaling, amino acid sensing and transport, and the initiation of protein translational initiation are of biological importance for the control of muscle mass. Elucidating the mechanisms coupling each of these events is limited in human in vivo studies and, therefore in vitro experiments are more appealing to uncover these mechanisms. However, for several substrates and hormones including insulin, there are discrepancies among concentrations observed in humans and those commonly used in cell culture studies to determine biological action and/or mechanism. From a clinical perspective, these studies may be limited in their applicability to human physiology. Therefore, the intent of this study was to examine the effect of a range of insulin concentrations on mTORC1 signaling in C2C12 myotubes and additionally, to determine whether the mRNA abundance of SLC38A2/SNAT2, SLC7A5/LAT1, SLC7A1/CAT1, and SLC36A1/PAT1 are dependent on mTORC1 signaling. Our data show for the first time that a low dose of insulin effectively stimulates downstream mTORC1 signaling and mRNA abundance of SLC38A2/SNAT2, SLC7A5/LAT1, and SLC36A1/PAT1. Furthermore, the addition of a low and high dose of rapamycin prevented an increase in SLC7A5/LAT1 mRNA abundance. These data provide insight into the role of insulin in the regulation of AAT mRNA abundance.

Examining the dose and duration of insulin on signal transduction in this study showed that insulin (0.5 nmol/L) stimulated a prolonged increase in Akt, mTOR, and ribosomal protein S6 phosphorylation whereas S6K1 and 4E‐BP1 phosphorylation were transiently increased. The transient increase in S6K1 and 4E‐BP1 remains unclear; however, it has been reported that increased cyclic adenosine monophosphate (cAMP) levels lead to phosphorylation of mTOR at multiple sites including Ser2448 and results in inhibition of downstream phosphorylation of S6K1 and 4E‐BP1 (Mothe‐Satney et al. 2004). Because cAMP is increased under ATP deficient conditions, it is plausible that prolonged exposure to insulin (without glucose) would increase cAMP levels leading to reduced phosphorylation of the downstream targets, S6K1 and 4E‐BP1. Collectively, these data demonstrate that insulin (0.5 nmol/L) effectively stimulates Akt phosphorylation and mTORC1 signaling activation in myotubes. To our knowledge, this is the first study to demonstrate mTORC1 activation with a low level of insulin as previous cell culture studies have shown changes (albeit to a larger magnitude) using higher levels of insulin (McDowell et al. 1998; Peyrollier et al. 2000; Kashiwagi et al. 2009; Liu et al. 2010; Luo et al. 2013).

The inhibitory actions of rapamycin on the mTOR signaling pathway are well established (Conejo and Lorenzo 2001; Shen et al. 2005; Luo et al. 2013). In a previous in vivo study conducted in our laboratory, 16 mg of rapamycin was administered to subjects that resulted in (1) plasma rapamycin concentrations of approximately 2.5 nmol/L and (2) inhibition of the amino acid‐induced upregulation of mTOR signaling and muscle protein synthesis (Dickinson and Rasmussen 2011). These data show that the level of rapamycin achieved in human subjects can effectively attenuate mTOR signaling. Subsequently, we examined the effect of this dose of rapamycin along with a higher dose of rapamycin on insulin‐stimulated Akt and mTOR signaling in C2C12 myotubes. Our results show that this (low) dose of rapamycin did not alter Akt, mTOR, and 4E‐BP1 phosphorylation. However, the lower dose of rapamycin reduced the insulin‐(0.5 nmol/L) stimulated increase in S6K1 and ribosomal protein S6 phosphorylation. Rommel et al. (2001) demonstrated a complete inhibition of S6K1 phosphorylation in C2C12 myotubes when administered 2 nmol/L rapamycin prior to the addition of insulin‐like growth factor I (albeit at a level of 10 ng/mL) for 15 min. Our initial rapamycin experiments were carried out for 30 min due to greater activation of downstream mTOR signaling components relative to 60 and 120 min. It is possible that further incubation with rapamycin would lead to changes in mTOR phosphorylation. Notably, the effects of high‐dose rapamycin increased Akt phosphorylation, which has been suggested as a feedback mechanism originating from downregulated S6K1 (Wan et al. 2007). Furthermore, mTOR was partially reduced while S6K1 and ribosomal protein S6 were completely inhibited by high rapamycin. On the contrary, phosphorylation of 4E‐BP1 was not reduced by high rapamycin indicating that a mTOR‐independent pathway may be responsible for the increase in 4E‐BP1 phosphorylation (Wang and Proud 2002; Thoreen et al. 2009). Our data are in line with previous reports demonstrating rapamycin attenuation of the insulin‐induced increase in mTOR downstream targets (Byfield et al. 2005; Shen et al. 2005; Luo et al. 2013), but also suggest that mTOR‐independent pathways may be playing a role in the activation of 4E‐BP1 and a potential feedback loop that leads to elevated phosphorylation of Akt.

AATs provide a crucial link between the availability of intracellular amino acids and protein metabolism. Notably, SNAT2 and LAT1 were shown to cooperate in order to increase intracellular leucine and stimulate mTORC1 kinase activity (Hyde et al. 2005; Evans et al. 2008; Drummond et al. 2010). Recent research has demonstrated that AAT are upregulated by amino acids in human muscle. For instance, Drummond et al. (2010) reported an increase in the mRNA abundance of SLC28A2/SNAT2, SLC7A5/LAT1, and PAT1/SLC36A1 1 h following ingestion of essential amino acids in young adults. In addition to amino acids, insulin has been shown to regulate protein metabolism and therefore it is conceivable that insulin may regulate the mRNA abundance of AAT and ultimately inward flux of amino acids. A number of studies have demonstrated the regulatory effects of insulin on AAT mRNA abundance. For example, SLC38A2/SNAT2 and SLC7A5/LAT1 mRNA abundance have been demonstrated to increase in L6 myotubes incubated with 100 nmol/L insulin for 30 min (Luo et al. 2013), in human trophoblast myotubes incubated with 1 nmol/L insulin for 4 h (Jones et al. 2010), and in L6 myotubes incubated with 100 nmol/L insulin for 8 h (Kashiwagi et al. 2009). Therefore, our goal was to incubate myotubes with a lower dose of insulin – such as seen in vivo in humans. We determined that after 4 h of insulin exposure, mRNA abundance of SLC38A2/SNAT2 and SLC7A5/LAT1 were elevated by 0.5 nmol/L insulin. Interestingly, in human skeletal muscle myotubes, mRNA abundance of SLC38A2/SNAT2 and SLC7A5/LAT1 was not changed after 30 min, 3 and 24 h of exposure to 100 nmol/L insulin (Gran and Cameron‐Smith 2011). Suryawan et al. (2013) demonstrated in neonatal pigs that SNAT2, SNAT3, LAT1, LAT2, PAT1, PAT2 protein abundance was unchanged when pigs were administered amino acids or insulin for 2 h. Similarly, we observed no changes in SLC38A2/SNAT2 and SLC7A5/LAT1 mRNA abundance prior to 4 h and this is likely due to the low dose of insulin (0.5 nmol/L) given in our study. In our study, we observed no changes in SLC7A1/CAT1 mRNA abundance after and up to 4 h of insulin exposure although SLC7A1/CAT1 mRNA abundance has been shown to be increased in cardiac myocytes after 24 h exposure to 100 nmol/L insulin (Simmons et al. 1996). CAT1 is an AAT that plays a role in arginine transport in which mRNA abundance of SLC7A1/CAT1 has been shown to be influenced by the addition of amino acids (Lopez et al. 2007). Our data suggest that the dose of 0.5 nmol/L insulin was insufficient to elevate mRNA abundance of SLC7A1/CAT1 or the response from SLC7A1/CAT1 mRNA is substantially delayed beyond 4 h when exposed to insulin. On the contrary, SLC36A1/PAT1 mRNA abundance, in our study, was elevated by 0.5 nmol/L insulin at 30 min and numerically elevated at 240 min. PAT1 has been suggested to be a crucial link in the activation of mTOR through PI3K/Akt signaling (Ogmundsdottir et al. 2012) and, therefore, it is possible that insulin could immediately affect regulation of SLC36A1/PAT1 mRNA abundance. Collectively, our data demonstrate that 0.5 nmol/L insulin stimulates mRNA abundance of the SLC38A2/SNAT2, SLC7A5/LAT1, and SLC36A1/PAT1.

Our next objective was to determine if rapamycin treatment could reduce the insulin‐stimulated increase in AAT mRNA abundance. Using two different concentrations of rapamycin (low and high), we show that low rapamycin does not prevent an increase in SLC38A2/SNAT2 mRNA abundance; however, a low dose of rapamycin was able to prevent the increase in the mRNA abundance of SLC7A5/LAT1 and, to some extent, SLC36A1/PAT1 mRNA. High rapamycin reduced mRNA abundance of SLC7A5/LAT1 and SLC36A1/PAT1 in addition to SLC38A2/SNAT2. Similar results in SNAT2 and LAT1 mRNA abundance have been demonstrated with high rapamycin when using higher levels of insulin in vitro (Adams 2007; Roos et al. 2009; Luo et al. 2013). Using RNAi, Rosario et al. (2013) showed that knockdown of mTORC1 or mTORC2 partially inhibited transport activity of SNAT2 and LAT1 in human trophoblasts whereas simultaneous knockdown of mTORC1 and mTORC2 completely inhibited transport activity. With high concentrations and/or prolonged exposure to rapamycin, it is suggested that mTORC2 activity and abundance becomes limited (Sarbassov et al. 2006). Based on our findings, it appears that mTORC1 may play a role in SLC7A5/LAT1 and perhaps SLC36A1/PAT1 transcription regulation. With the lack of amino acids in this study, it is conceivable that the general control nonrepressed (GCN2) pathway may be involved in the regulation of AAT mRNA abundance; however, a recent report indicated that the regulation of AAT by insulin involves a GCN2‐independent pathway (Luo et al. 2013).

In summary, we specifically demonstrate that a low concentration of insulin effectively activates Akt and mTORC1 signaling. Insulin‐induced mTORC1 signaling is reduced by exposure to a high‐rapamycin dose and by a smaller dosage used in human studies. Furthermore, 0.5 nmol/L insulin increases the mRNA abundance of SLC38A2/SNAT2, SLC7A5/LAT1, and SLC36A1/PAT1. The insulin‐induced increase in SLC7A5/LAT1 mRNA abundance is prevented with the addition of a low (and high) dose of rapamycin. Collectively, we report here two novel findings that (1) a low insulin concentration (similar to in vivo postprandial levels) increases mTORC1 signaling and SLC38A2/SNAT2, SLC7A5/SLC7A5/LAT1, and SLC36A1/PAT1 mRNA abundance; and (2) the insulin‐induced increase in SLC7A5/LAT1 mRNA abundance is mTORC1 dependent.

## Conflict of Interest

None declared.
